# Performance Improvement of Near-Infrared Spectroscopy-Based Brain-Computer Interface Using Regularized Linear Discriminant Analysis Ensemble Classifier Based on Bootstrap Aggregating

**DOI:** 10.3389/fnins.2020.00168

**Published:** 2020-03-04

**Authors:** Jaeyoung Shin, Chang-Hwan Im

**Affiliations:** ^1^Department of Electronic Engineering, Wonkwang University, Iksan, South Korea; ^2^Department of Biomedical Engineering, Hanyang University, Seoul, South Korea

**Keywords:** brain-computer interface, bootstrap aggregating, ensemble learning, near-infrared spectroscopy, pattern classification

## Abstract

Ensemble classifiers have been proven to result in better classification accuracy than that of a single strong learner in many machine learning studies. Although many studies on electroencephalography-brain-computer interface (BCI) used ensemble classifiers to enhance the BCI performance, ensemble classifiers have hardly been employed for near-infrared spectroscopy (NIRS)-BCIs. In addition, since there has not been any systematic and comparative study, the efficacy of ensemble classifiers for NIRS-BCIs remains unknown. In this study, four NIRS-BCI datasets were employed to evaluate the efficacy of linear discriminant analysis ensemble classifiers based on the bootstrap aggregating. From the analysis results, significant (or marginally significant) increases in the bitrate as well as the classification accuracy were found for all four NIRS-BCI datasets employed in this study. Moreover, significant bitrate improvements were found in two of the four datasets.

## Introduction

In general, brain-computer interface (BCI) systems (1) measure the brain signals in response to specific stimuli or mental tasks, (2) extract representative features from the acquired brain signals, (3) translate them by applying pattern recognition algorithms, and (4) control external devices or communicate with environments (Wolpaw et al., 2002; Schalk et al., 2004). In some cases, feedbacks are given to BCI users to improve the BCI performance (Lebedev and Nicolelis, 2006; Hwang et al., 2009; Kanoh et al., 2009; Blankertz et al., 2010). Among the aforementioned procedures, feature selection and pattern recognition are the most important parts that determine the overall performance of a BCI system (Nicolas-Alonso and Gomez-Gil, 2012). Particularly in the case of near-infrared spectroscopy (NIRS)-BCI, many different kinds of features have been tested to validate their suitability to various NIRS-BCI systems with different experimental paradigms and environments (Hwang et al., 2014; Naseer et al., 2016). It was reported that the temporal mean, maximum, and slope yielded reasonable BCI performance (Naseer and Hong, 2015); however, there is no consensus on the most suitable features that can be generally applied to different NIRS-BCIs. In addition, various kinds of pattern recognition methods have also been proposed and tested with the aim to improve the performance of NIRS-BCI systems. Among them, linear discriminant analysis (LDA) classifier has been most widely used for NIRS-BCIs because of its excellent performance reflected by both a fast learning rate and a good classification performance (Holper and Wolf, 2011; Power et al., 2011, 2012a,b; Schudlo and Chau, 2014; Hong et al., 2015; Shin et al., 2016, 2018a; Hong and Khan, 2017). In applying the classifier, dimension reduction or feature selection methods are generally employed because the number of NIRS feature vectors is usually larger than that of training datasets and this might degrade the BCI performance due to the poor empirical sample covariance (Hwang et al., 2016; Hong et al., 2018; Sereshkeh et al., 2019). Regularization with a shrinkage parameter can be another option to alleviate the adverse effect of the large dimensionality (Fazli et al., 2012).

Ensemble learning can be considered a good substitute for further improving the overall BCI performance. Ensemble classifiers are grounded in the theory that a combination of multiple weak learners that barely exceed the chance level is capable of achieving better classification accuracy than that of a single strong leaner. It has been reported that ensemble classifiers can improve the performance of electroencephalography-BCIs. Sun et al. (2007); Ahangi et al. (2013), and Gao et al. (2016) employed various types of ensemble learning methods, e.g., bagging, boosting, and random subspace, etc., to evaluate the feasibility of ensemble learning for motor imagery EEG data. Fatourechi et al. (2008) stacked support vector machine (SVM) classifiers to classify finger flexion movement with a low false positive rate. Rakotomarnonjy and Guigue (2008) employed a majority voting system based on SVM for P300 signals by an oddball paradigm. Hassan and Bhuiyan (2017) demonstrated the automated identification of sleep stages by means of boosting methods, and Hosseini et al. (2018) exploited random subspace ensemble and majority voting for seizure detection. In the case of NIRS-BCIs, there have been a few studies that employed ensemble classifiers (Schudlo and Chau, 2015; Gurel et al., 2019), but they did not compare the performance of ensemble classifiers with that of conventional classifiers. To the best of our knowledge, no study has systematically investigated the performance improvement of NIRS-BCIs by the employment of ensemble classifiers. Specifically, because regularized linear discriminant of analysis (RLDA) alleviating the degradation of classification accuracy is generally known to be appropriate for the high dimensional NIRS dataset, we employed RLDA as a type of weak learner in the ensemble method. In the present study, for the first time, we explore whether the performance of NIRS-BCIs can be enhanced by using an ensemble of weak learners rather than a single strong learner through a systematic comparison of BCI performances with multiple NIRS datasets recorded with different experimental paradigms and/or under different recording environments.

## Materials and Methods

We employed four different NIRS datasets recorded by the first author of this paper. Datasets denoted by “dataset I” and “dataset II” can be freely downloaded at: http://doc.ml.tu-berlin.de/hBCI/ (Shin et al., 2017b) and “dataset III” can be downloaded at: http://dx.doi.org/10.14279/depositonce-5830 (Shin et al., 2018c). “Dataset IV” is a NIRS dataset used in the study of Shin et al. (2018b). All data processing was performed using MATLAB R2018b (Mathworks, MA, United States) and the BBCI toolbox^1^ (Blankertz et al., 2016). A brief summary of the datasets I–IV is given in Table 1.

**TABLE 1:** Summary of the four datasets employed in this study.

**Datasets**	**Duration of task period (s)**	**Post-task break (s)**	**The number of trials per task**	**Types of mental tasks**
Dataset I	10	15–17	30	MI tasks (left- and right-hand grasping)
Dataset II	10	15–17	30	MA and IS
Dataset III	10	13–15	30	WG and IS
Dataset IV	10	16–18	30	MI (right-hand finger tapping), MA, and IS

*MI, MA, WG, and IS represent mental imagery, mental arithmetic, word generation, and idle state, respectively.*

### Datasets I and II

#### Data Recording

**FIGURE 1:**
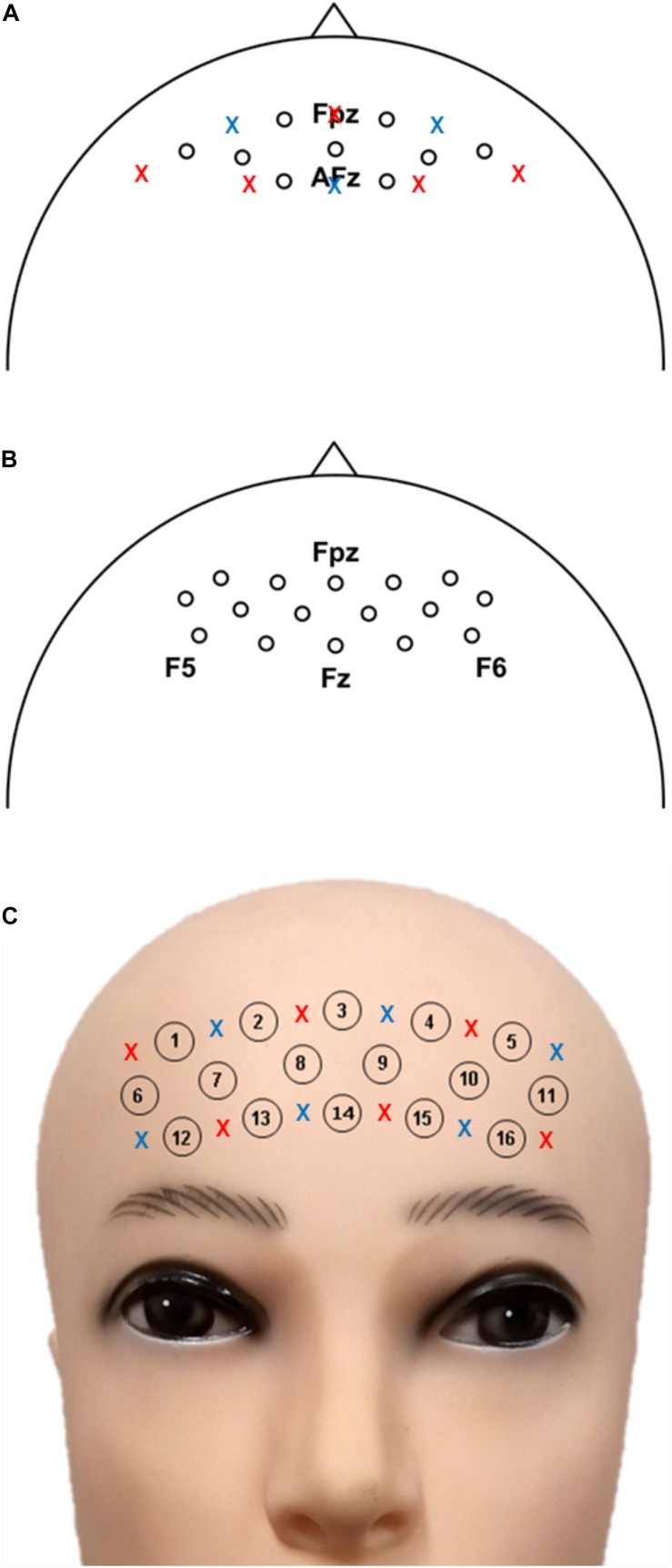
Location of the NIRS channels for **(A)** datasets I-II, **(B)** dataset III, and **(C)** dataset IV. These figures were amended from Shin et al. (2017b; 2018b; 2018c). Blue and red crosses are the specific positions of source and detector, respectively. The specific source and detector positions for dataset II are not available.

Near-infrared spectroscopy data were collected using NIRScout (NIRx GmbH, Berlin, Germany) at a sampling rate of 12.5 Hz. Adjacent source-detector distance was fixed to 30 mm. The locations of nine physical NIRS channels over the prefrontal area are depicted in Figure 1A.

#### Two Motor Imagery Tasks (Dataset I)

Twenty-nine participants were seated and performed two designated motor imagery (MI) tasks (kinesthetic motor imagery of grasping with either the left or the right hand at a rate of approximately 1 Hz) during the task period (0–10 s), 30 times each, in a randomized order.

#### Mental Arithmetic vs. Idle State (Dataset II)

The same participants who participated in the previously described MI experiment (dataset I) were asked to perform a mental arithmetic (MA) task. Starting with an initial problem of subtraction of a single digit between 6 and 9 from a three-digit number (e.g., 219 – 7), they continuously subtracted the given single-digit number from the result of the former calculation (e.g., 219 – 7 = 212, 212 – 7 = 205, 205 – 7 = 198,…) as fast as they could during the task period (0–10 s). For the idle state (IS), the participants relaxed and tried not to come up with any distractive thoughts during the task period (0–10 s). The MA and IS tasks were randomly repeated 30 times each.

### Dataset III

#### Data Recording

Near-infrared spectroscopy data were acquired with NIRScout at a sampling rate of 10.4 Hz. Sixteen sources and 16 detectors were placed over the frontal area (around AFz), and sixteen NIRS channels with a source-detector separation of 30 mm were created. The NIRS channel locations are illustrated in Figure 1B.

#### Word Generation vs. Idle State

For the word generation (WG) task, twenty-six participants were seated and kept coming up with words beginning with a randomly given syllable as quickly as they could during the given task period (0–10 s). Repetition of the same word was not allowed for each trial to avoid potential adaptation. For the IS, the participants took a rest and tried not to think about anything for 10 s. The WG and IS tasks were randomly performed 30 times each.

### Dataset IV

#### Data Recording

Near-infrared spectroscopy data were sampled at a sampling rate of 13.3 Hz using a portable NIRS acquisition system (LIGHTNIRS, Shimadzu Corp., Kyoto, Japan). Six sources and six detectors over the prefrontal area created 16 NIRS channels with a 30-mm source-detector separation. The locations of the 16 physical NIRS channels are illustrated in Figure 1C.

#### Mental Arithmetic vs. Motor Imagery vs. Idle State

For the MI task, seventeen participants were seated and imagined complex finger tapping at a rate of approximately 2 Hz for 10 s. The participants performed the MA task in the same way as with the dataset II, and for the IS, they relaxed without performing any specific mental task. The MI, MA, and IS tasks were randomly performed 30 times each.

### Behavioral Data

Available behavioral data are stored in each repository for the datasets I–IV.

### Preprocessing

In the original articles (Shin et al., 2017b, 2018b,c), the four datasets were preprocessed in different manners. For the sake of fair performance comparison, all datasets were preprocessed in the same manner. The hemodynamic changes in reduced and oxidized hemoglobin (ΔHbR and ΔHbO) were converted from the raw light intensity changes using the modified Beer–Lambert law, and were then band-pass filtered using a zero-phase Butterworth filter with a passband of 0.01–0.09 Hz to eliminate physiological noises (Matthews et al., 2008). Any trials were not excluded because the recorded data were minimally affected by motion artifacts.

### Classification

The classification procedures were performed using the data from each of the participants separately.

#### Features

The baseline of the filtered data was corrected by subtracting the temporal mean of the data within [-1 0] s interval. The baseline-corrected data were then segmented to epochs ranging from 0 to 15 s, which contained part of the post-task break period, considering the hemodynamic delay in the order of several seconds (approximately 6–8 s) (Cui et al., 2010). Feature vectors consisted of the temporal mean values of ΔHbR and ΔHbO within two windows of [5 10] and [10 15] s. The number of features was [the number of NIRS channels] × [the number of NIRS chromophores (2)] × [the number of windows (2)].

#### Single Strong Learner

Three types of classifiers were considered, namely SVM, LDA, and RLDA. For SVM, the linear kernel was employed and the feature vectors were standardized by subtracting mean and dividing by standard deviation. Other parameters were default options given by MATLAB. For LDA, typical LDA was used. Normally, typical LDA classifier find the *k*^th^class which maximize $\log\pi_{k}-\frac{1}{2}\,\mu_{k}^{T}\,{\text{Σ}}^{-1}\,\mu_{k}+x^{T}\,{\text{Σ}}^{-1}\,\mu_{k}$, where π_*k*_, μ_*k*_, and Σ are the *a prior* probability and the mean of samples in the *k*^th^class, and the covariance matrix common to all classes, respectively. However, In the case of NIRS feature vectors, typical LDA is not likely to be adequate because of the degradation of classification accuracy due to the high-dimensionality, in other words, the number of features is greater than the number of samples. That is a reason why the RLDA classifier with a shrinkage parameter (γ) was employed to alleviate the adverse effects of large dimensionality on the BCI performance by replacing the empirical covariance matrix Σ with (1-γ)Σ + γ*I*, where *I* is the identity matrix. The optimal γ between 0 and 1 was determined individually based on the Ledoit and Wolf (2004), Schäfer and Strimmer (2005); Blankertz et al. (2011); Lemm et al. (2011). For the ternary classification, linear SVM and LDA with “one-versus-one” error-correcting output model were used, and the multi-class RLDA were applied.

#### Ensemble of Weak Learners

The bootstrap aggregating (Bagging) algorithm subsamples *N*_learn_ training sets of the same size with replacement (fraction of the training set to resample for every weak learner: 100% in this study), then builds *N*_learn_ classification models for each training set using a weak learner *h*(⋅). The final aggregate classification model based on a majority voting *H*(*x*) is given by:

(1) $$H\,\left(x\right)=\mathrm{sign}\,\left(\sum_{n=1}^{N_{l\,e\,a\,r\,n}}\text{sign}\,\left(h_{n}\,\left(x\right)\right)\right)$$

To verify the efficacy of LDA classifier, RLDA classifier was used as a weak learner and the value of λ was set to 0.1 as a rule of thumb. Stratified random sampling was applied to split the whole dataset into ten subsets, and a 10 × 10-fold cross-validation was performed for both the single strong learner and the ensemble of weak learners, resulting in the “strong classification accuracy (*acc*_strong_)” and the “Bagging classification accuracy (*acc*_bag_),” respectively.

### Bitrate

Information transfer rate (ITR) is one of the most popular metrics to evaluate the performance of communication systems. The ITR per minute, called bitrate, is utilized to assess the performance of BCI systems, as follows (Dornhege et al., 2007):

$$bitrate=\frac{60}{T}\cdot [{\text{log}}_{2}\left(n\right)+acc\cdot {\text{log}}_{2}\left(acc\right)+$$

(2) $$\left(1-acc\right)\cdot {\text{log}}_{2}\left(\frac{1-a\,c\,c}{n-1}\right)](bits/min),$$

where *T*, *n*, and *acc* are a single trial length (usually the length of the task period), the number of different types of mental tasks, and classification accuracy, respectively.

### Statistical Test

Normality of data distribution was tested with Anderson–Darling test, and according to the test decision (*p* < 0.05), two-tailed paired *t*-test was performed to test the hypothesis that the average of *acc*_bag_ and *acc*_strong_ are different. The *p*-values were corrected by false positive rate (Benjamini and Yekutieli, 2001) unless otherwise noted.

## Results

### Classification Accuracy

**FIGURE 2:**
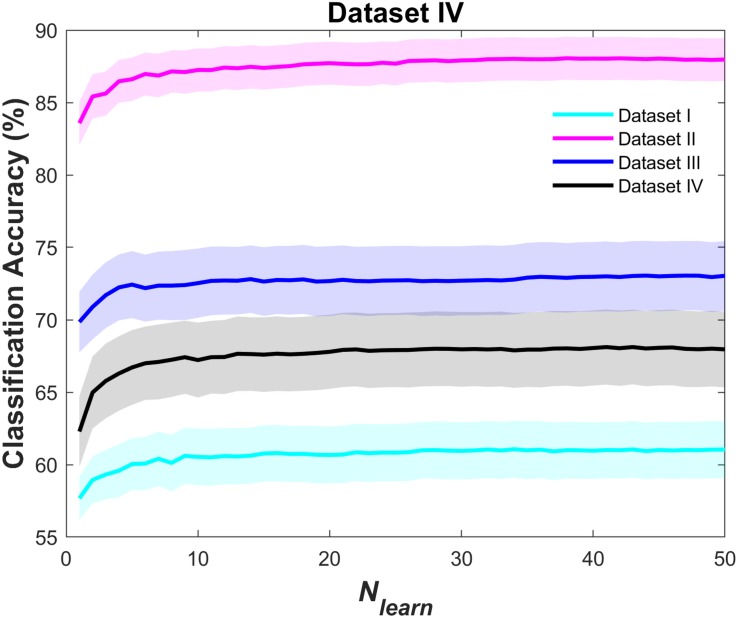
Grand average of *acc*_bag_ as a function of *N*_learn_ for dataset I (cyan), dataset II (magenta), dataset III (blue), and dataset IV (black). The shaded area represents the standard error of the mean.

**FIGURE 3:**
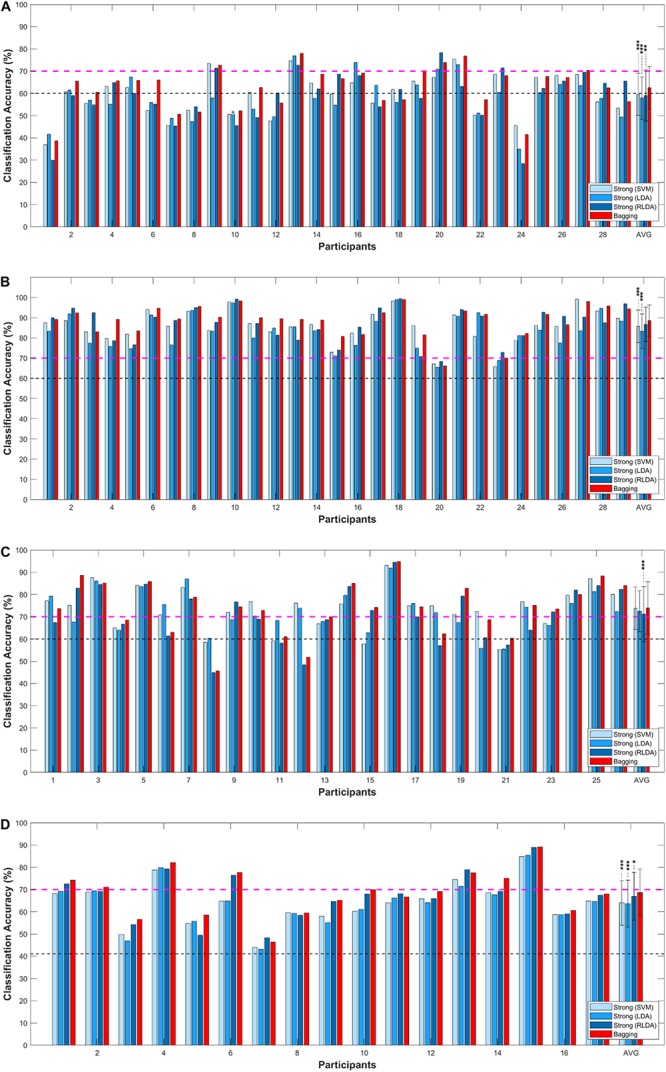
Comparisons of individual *acc*_strong_ (blue) and *acc*_bag_ (red) for **(A)** dataset I, **(B)** dataset II, **(C)** dataset III, and **(D)** dataset IV. The error-bar indicates the standard deviation. The magenta dashed line represents the effective BCI threshold level (70.0%) indicating (Vidaurre and Blankertz, 2010). AVG represents the average of the classification accuracies across all participants. ^∗^Corrected-*p* < 0.05, ^∗∗^corrected-*p* < 0.01, and ^∗∗∗^corrected-*p* < 0.001 (false discovery rate correction).

**FIGURE 4:**
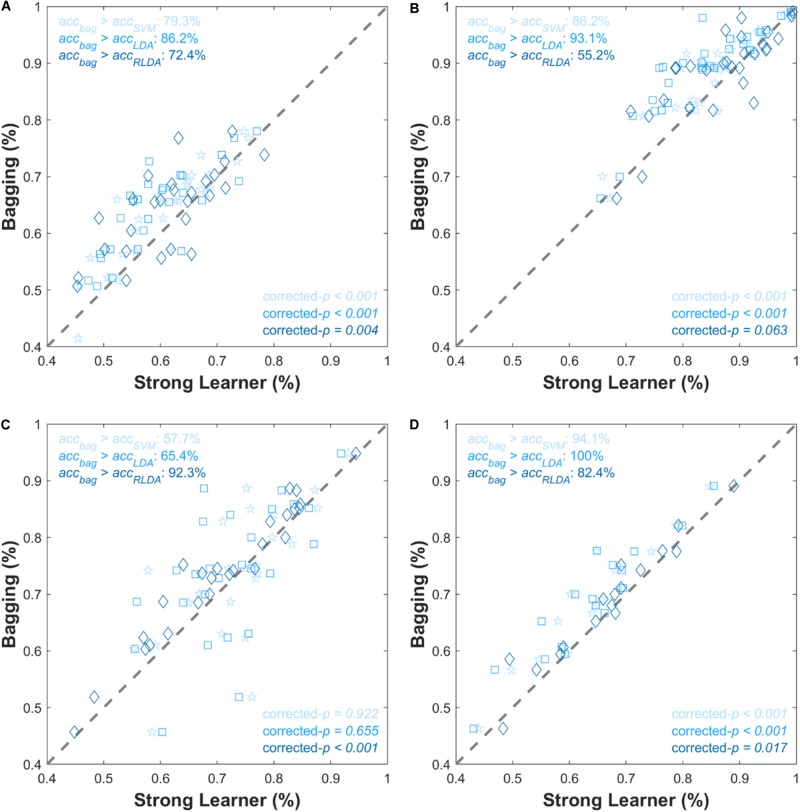
Scatter plots comparing individual classification accuracies for **(A)** dataset I, **(B)** dataset II, **(C)** dataset III, and **(D)** dataset IV. The *x*- and *y*-axes correspond to *acc*_strong_ and *acc*_bag_, respectively. Gray dashed lines are points where *acc*_strong_ = *acc*_bag_. The corrected-*p*-values represent the significance of improvement of the classification accuracy by the bagging method. Pentagram, square, and diamond symbols are for SVM, LDA, and RLDA, respectively. Symbol color is in accordance with the bar color shown in Figure 3.

Figure 2 shows the grand average of the classification accuracy as a function of *N*_learn_. As the *N*_learn_ increased, the classification accuracies improved irrespective of the type of NIRS datasets. Overall, the rate of increment rapidly decreased where *N*_learn_ > 10, and then the classification accuracy was almost converged where *N*_learn_ = 50. Comparisons of individual *acc*_strong_and *acc*_bag_ are presented in Figures 3, 4. In Figure 3, magenta dashed lines indicate the classification accuracy value (70%) generally known as a threshold for effective BCI control (Vidaurre and Blankertz, 2010). Black dashed lines denote the theoretical chance levels based on binomial distribution (*p* < 0.05) (Combrisson and Jerbi, 2015). For Figures 3, 4, the values of *N*_learn_ to compute *acc*_bag_ are individually different and the optimal values of *N*_learn_ were chosen in the range of 10 ≤*N*_learn_≤ 50. For the dataset I, the grand average of *acc*_bag_ (62.6 ± 9.6%) was significantly higher than the averages of all three *acc*_strong_ (i.e., *acc*_SVM_ (59.6 ± 9.5%), *acc*_*LDA*_ (57.9 ± 9.5%), and *acc*_RLDA_ (59.1 ± 11.6%). For the dataset II, apart from the *acc*_RLDA_ (86.7 ± 8.6%), the grand average of *acc*_bag_ (88.5 ± 7.7%) was significantly higher than others. The grand average of *acc*_bag_ (74.8 ± 11.8%) for the dataset III was not significantly higher apart from the grand average of *acc*_RLDA_ (71.2 ± 12.4%) but the others. The bagging algorithm yielded the significant difference of ternary classification accuracy (71.2 ± 12.4%) compared to the other strong learners. The individual classification accuracies are provided in digits in the Supplementary Information. In Figure 4, symbols above the dashed diagonal line represent that the Bagging algorithm is more advantageous to improve the individual classification accuracy (i.e., *acc*_bag_ > *acc*_strong_), while those below the diagonal line represent that *acc*_bag_ < *acc*_strong_. It was revealed that *acc*_bag_ exceeded significantly *acc*_strong_ in nine cases out of 12 comparisons (*p* < 0.05). For the ternary classification (dataset IV), the improvement of bitrate was particularly significant in all cases.

### Bitrate

**FIGURE 5:**
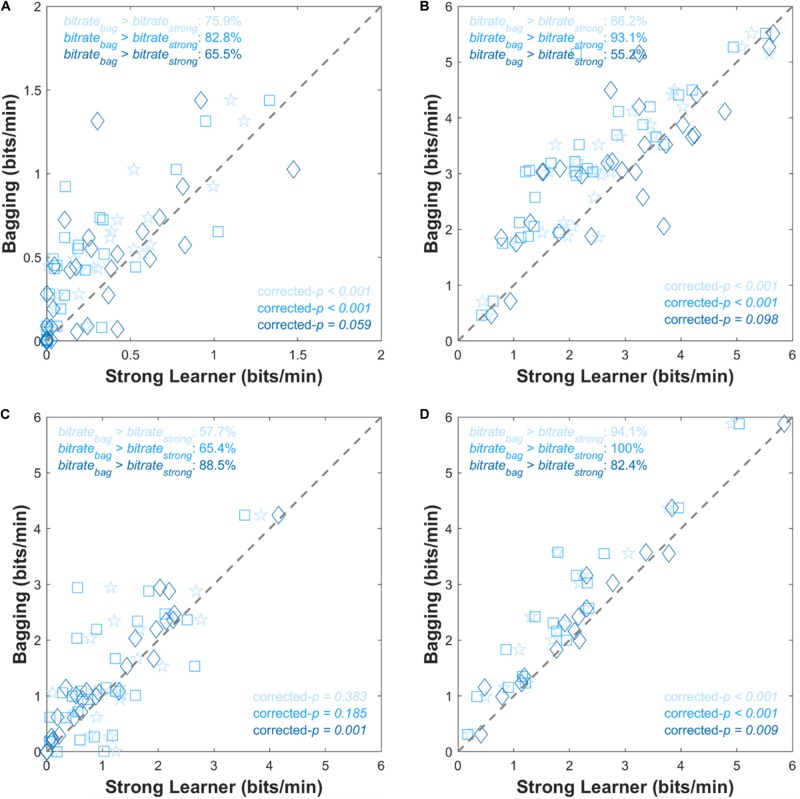
Scatter plots comparing individual bitrates for **(A)** dataset I, **(B)** dataset II, **(C)** dataset III, and **(D)** dataset IV. The *x*- and *y*-axes correspond to *bitrate*_strong_ and *bitrate*_bag_, respectively. Gray dashed lines are points where *bitrate*_strong_ = *bitrate*_bag_. The corrected-*p*-values represent the significance of improvement of the bitrate by the bagging method. Pentagram, square, and diamond symbols are for SVM, LDA, and RLDA, respectively. Symbol color is in accordance with the bar color shown in Figure 3.

Figure 5 shows comparisons of individual bitrates (bits/min), where the symbols above the dashed diagonal line represent that the Bagging algorithm resulted in higher bitrates than the single strong learner, and vice versa. In the case of the dataset I, significant (or marginally significant) bitrate improvements were observed and it was observed in over 70% of individual results in all three cases. The bagging highly significantly improved bitrates when it comes to the comparisons versus SVM or LDA (corrected-*p* < 0.001). For the dataset III, The bagging was significantly superior to RLDA when it came to bitrates (corrected-*p* < 0.001) unlikely the rest two cases. Note that the bagging always outperformed typical LDA in the ternary system (dataset IV).

## Discussion

### Summary

In this study, we explored, for the first time, whether the performance of binary and ternary NIRS-BCI systems can be improved by using ensemble classifiers. We created ensembles of weak learners based on the Bagging algorithm. Four NIRS-BCI datasets recorded with different experimental paradigms were used for the quantitative performance comparisons between the Bagging algorithm and the conventional single stronger learner approach. Our results demonstrated that the Bagging algorithm significantly (or marginally significantly) outperformed the single strong learner in terms of classification accuracy and bitrate in all the cases of datasets.

### Necessity of Using an Appropriate Ensemble Classifier

To create a better ensemble classifier, it is important to select an appropriate ensemble aggregation method, that is, a type of weak learner, and to determine the optimal hyperparameters, such as the number of ensemble learning cycles (*N*_learn_ in this study). For the optimization of the hyperparameters, various approaches can be employed, such as a grid search, random search (Bergstra and Bengio, 2012), and the Bayesian optimization (Mockus, 2012); however, since the optimized hyperparameters are generally dependent on the test set employed in the optimization, it is practically difficult to derive universally optimized hyperparameters. This implies that simply using ensemble classifiers does not always guarantee an enhanced performance in NIRS-BCIs and that a customized ensemble classifier appropriate for the given datasets needs to be employed. If the aggregation method and hyperparameters are not properly chosen or determined based on subjective assumptions, desired results might hardly be obtained. For example, when a binary decision tree was arbitrarily designated as a weak learner in this study, the classification accuracy was not enhanced at all compared to *acc*_strong_. In addition, as shown in Figure 2, small values of *N*_learn_ resulted in low *acc*_bag_, even lower than *acc*_strong_ because the small size of ensembles was not able to be trained sufficiently with various sample sets, causing to deteriorate classification accuracy. On the other hand, the bagging ensemble containing enough weak learners reduced effectively variance of estimates, which is consistent with the bagging ensemble theoretical background (Mayr et al., 2014). As mentioned above, the γ value for a weak learner was chosen as a rule of thumb. By changing the γ value from 0.001 to 0.5, in addition, we assessed whether the improvement of classification accuracy was possible. As a result, γ = 0.1 yielded significant difference in classification accuracies against *N*_learn_ (Bonferroni corrected-*p* < 0.001, not shown in the text) except the dataset I. This fact underpins the importance of proper parameter selection regarding ensemble learning methods as well. In this study, we could successfully achieve an enhanced BCI performance by using RLDA classifier with appropriate hyperparameters (10 ≤*N*_learn_≤ 50 and γ = 0.1).

### Limitation: Bitrate and Real Time Analysis

We improved the bitrate by successfully improving the classification accuracy in the present study. However, it is very difficult to reduce the trial length due to the inherent limitations of fNIRS-BCIs, such as slow response time due to hemodynamic delay. Recently, steady-state visually evoked potential (SSVEP)-BCI has shown the average performance of 701 bit/min (Nagel and Spüler, 2019). Even though many efforts have been devoted to improving the bitrate of fNIRS-BCIs (Cui et al., 2010; Zafar and Hong, 2017; Hong and Zafar, 2018), it is difficult to bridge the performance gap between fNIRS-BCIs and EEG-BCIs. However, for such SSVEP-BCI which is a type of exogenous BCIs, the need for an external stimulus causing user fatigue easily could be problematic.

This study dealt with the efficacy of the ensemble learning methods using the previously released open-access NIRS-BCI datasets. Since the experimental environment and analysis techniques for the implementation of real-time NIRS-BCIs are completely different from those for the implementation of offline NIRS-BCIs, it does not make sense to verify the feasibility of ensemble learning for online NIRS-BCIs with the offline NIRS-BCI datasets. Therefore, the efficacy of ensemble learning for online NIRS-BCIs should be validated in the future studies.

### Efforts to Improve the Performance of NIRS-BCIs: Future Perspective

There have been many efforts to improve the overall performance of NIRS-BCIs. Recently, off-the-shelf NIRS systems adopting novel designs and form factors have been introduced to the market and their usefulness in NIRS-BCIs has been verified (Shin et al., 2017a; Kim et al., 2018; Kwon et al., 2018; Lancia et al., 2018). However, most of the new form factors adopted by the recent NIRS systems do not possess general applicability because they are designed to record hemodynamic changes from the prefrontal area only. In addition, artificial intelligence methods based on deep learning have demonstrated their potential in enhancing the performance of BCI systems (Cecotti and Graser, 2011; Chiarelli et al., 2018; Lawhern et al., 2018; Nicholas et al., 2018; Sakhavi et al., 2018). Even though some studies have reported the superiority of the deep learning-based approach compared to the conventional machine learning methods (Trakoolwilaiwan et al., 2018), there still exist controversies regarding the employment of these opinions (Hennrich et al., 2015). Since deep learning techniques generally depend on human factors, such as how well the deep learning model structure is designed, objective and thorough investigations of deep learning models that can enhance the performance of NIRS-BCIs are necessary. Conversely, some recent studies showed the potential of the incorporated use of ensemble learning concepts with deep learning approaches (Xiao et al., 2018). The development of a novel ensemble classifier incorporated with deep learning techniques and its application to NIRS-BCIs would be a promising topic, which we would like to pursue in future studies.

## Conclusion

In this study, we demonstrated the effect of performance enhancement of NIRS-BCIs by the employment of a proper ensemble classifier, the RLDA ensemble classifier is based on the Bagging algorithm in this study, which has never been investigated before. As a result, the ensemble learning method employed was beneficial to improve the classification accuracies of all four datasets considered in this study. In our future studies, the ensemble classifier introduced in this study would be applied to new NIRS-BCI datasets to confirm its general availability, and new types of ensemble classifiers that can further enhance the performance of NIRS-BCI would also be tested.

## Data Availability Statement

Publicly available datasets were analyzed in this study. This data can be found here: http://dx.doi.org/10.14279/depositonce-5830, http://doc.ml.tu-berlin.de/hBCI/, and https://doi.org/10.6084/m9.figshare.9198932.

## Ethics Statement

Ethical review and approval was not required for the study on human participants in accordance with the local legislation and institutional requirements. Written informed consent for participation was not required for this study in accordance with the national legislation and the institutional requirements.

## Author Contributions

JS planned the study and analyzed the data. C-HI supervised the work. Both authors wrote and reviewed the manuscript.

## Conflict of Interest

The authors declare that the research was conducted in the absence of any commercial or financial relationships that could be construed as a potential conflict of interest. The reviewer JM declared a past co-authorship with one of the authors JS to the handling Editor.

## Footnotes

1 https://github.com/bbci/bbci_public

## References

[B1] Ahangi A. Karamnejad M. Mohammadi N. Ebrahimpour R. Bagheri N. (2013). Multiple classifier system for EEG signal classification with application to brain-computer interfaces. *Neural Comput. Appl.* 23 1319–1327. 10.1109/TBME.2013.2248153 23446029

[B2] Benjamini Y. Yekutieli D. (2001). The control of the false discovery rate in multiple testing under dependency. *Ann. Stat.* 29 1165–1188. 10.1186/1471-2105-9-114 18298808 PMC2375137

[B3] Bergstra J. Bengio Y. (2012). Random search for hyper-parameter optimization. *J. Mach. Learn. Res.* 13 281–305.

[B4] Blankertz B. Acqualagna L. Dähne S. Haufe S. Schultze-Kraft M. Sturm I. (2016). The berlin brain-computer interface: progress beyond communication and control. *Front. Neurosci.* 10:530. 10.3389/fnins.2016.00530 27917107 PMC5116473

[B5] Blankertz B. Lemm S. Treder M. Haufe S. Müller K.-R. (2011). Single-trial analysis and classification of ERP components—a tutorial. *Neuroimage* 56 814–825. 10.1016/j.neuroimage.2010.06.048 20600976

[B6] Blankertz B. Tangermann M. Vidaurre C. Fazli S. Sannelli C. Haufe S. (2010). The Berlin brain–computer interface: non-medical uses of BCI technology. *Front. Neurosci.* 4:00198. 10.3389/fnins.2010.00198 21165175 PMC3002462

[B7] Cecotti H. Graser A. (2011). Convolutional neural networks for P300 detection with application to brain-computer interfaces. *IEEE Trans. Pattern Anal. Mach. Intell.* 33 433–445. 10.1109/TPAMI.2010.125 20567055

[B8] Chiarelli A. M. Croce P. Merla A. Zappasodi F. (2018). Deep learning for hybrid EEG-fNIRS brain-computer interface: application to motor imagery classification. *J. Neural Eng.* 15:036028. 10.1088/1741-2552/aaaf82 29446352

[B9] Combrisson E. Jerbi K. (2015). Exceeding chance level by chance: the caveat of theoretical chance levels in brain signal classification and statistical assessment of decoding accuracy. *J. Neurosci. Methods* 250 126–136. 10.1016/j.jneumeth.2015.01.010 25596422

[B10] Cui X. Bray S. Reiss A. L. (2010). Speeded near infrared spectroscopy (NIRS) response detection. *PLoS One* 5:15474. 10.1371/journal.pone.0015474 21085607 PMC2978722

[B11] Dornhege G. Millán J. R. Hinterberger T. Mcfarland D. Müller K.-R. (2007). *Toward Brain-Computer Interfacing.* Cambridge, MA: MIT press.

[B12] Fatourechi M. Ward R. K. Birch G. E. (2008). A self-paced brain-computer interface system with a low false positive rate. *J. Neural Eng.* 5 9–23. 10.1088/1741-2560/5/1/002 18310807

[B13] Fazli S. Mehnert J. Steinbrink J. Curio G. Villringer A. Müller K.-R. (2012). Enhanced performance by a hybrid NIRS-EEG brain computer interface. *Neuroimage* 59 519–529. 10.1016/j.neuroimage.2011.07.084 21840399

[B14] Gao L. Cheng W. Zhang J. H. Wang J. (2016). EEG classification for motor imagery and resting state in BCI applications using multi-class Adaboost extreme learning machine. *Rev. Sci. Instrum.* 87:085110. 10.1063/1.4959983 27587163

[B15] Gurel N. Z. Jung H. Hersek S. Inan O. T. (2019). Fusing near-infrared spectroscopy with wearable hemodynamic measurements improves classification of mental stress. *IEEE Sens. J.* 19 8522–8531. 10.1109/jsen.2018.2872651 PMC7731966 33312073

[B16] Hassan A. R. Bhuiyan M. I. H. (2017). Automated identification of sleep states from EEG signals by means of ensemble empirical mode decomposition and random under sampling boosting. *Comput. Methods Programs Biomed.* 140 201–210. 10.1016/j.cmpb.2016.12.015 28254077

[B17] Hennrich J. Herff C. Heger D. Schultz T. (2015). “Investigating deep learning for fNIRS based BCI,” in *37th International Conference of the IEEE Engineering in Medicine and Biology Society (EMBC)*, (Milan), 2844–2847. 10.1109/EMBC.2015.7318984 26736884

[B18] Holper L. Wolf M. (2011). Single-trial classification of motor imagery differing in task complexity: a functional near-infrared spectroscopy study. *J. Neuroeng. Rehabi.* 8:34. 10.1186/1743-0003-8-34 21682906 PMC3133548

[B19] Hong K.-S. Khan M. J. (2017). Hybrid brain-computer interface techniques for improved classification accuracy and increased number of commands: a review. *Front. Neurorobot.* 11:35. 10.3389/fnbot.2017.00035 28790910 PMC5522881

[B20] Hong K.-S. Khan M. J. Hong M. J. (2018). Feature extraction and classification methods for hybrid fNIRS-EEG brain-computer interfaces. *Front. Hum. Neurosci.* 12:246. 10.3389/fnhum.2018.00246 30002623 PMC6032997

[B21] Hong K.-S. Naseer N. Kim Y.-H. (2015). Classification of prefrontal and motor cortex signals for three-class fNIRS-BCI. *Neurosci. Lett.* 587 87–92. 10.1016/j.neulet.2014.12.029 25529197

[B22] Hong K. S. Zafar A. (2018). Existence of initial dip for BCI: an illusion or reality. *Front. Neurorobot.* 12:69. 10.3389/fnbot.2018.00069 30416440 PMC6212489

[B23] Hosseini M. P. Pompili D. Elisevich K. Soltanian-Zadeh H. (2018). Random ensemble learning for EEG classification. *Artif. Intell. Med.* 84 146–158. 10.1016/j.artmed.2017.12.004 29306539

[B24] Hwang H. J. Choi H. Kim J. Y. Chang W. D. Kim D. W. Kim K. W. (2016). Toward more intuitive brain-computer interfacing: classification of binary covert intentions using functional near-infrared spectroscopy. *J. Biomed. Opt.* 21:091303. 10.1117/1.JBO.21.9.091303 27050535

[B25] Hwang H.-J. Kwon K. Im C.-H. (2009). Neurofeedback-based motor imagery training for brain–computer interface (BCI). *J. Neurosci. Meth.* 179 150–156. 10.1016/j.jneumeth.2009.01.015 19428521

[B26] Hwang H.-J. Lim J.-H. Kim D.-W. Im C.-H. (2014). Evaluation of various mental task combinations for near-infrared spectroscopy-based brain-computer interfaces. *J. Biomed. Opt.* 19:077005. 10.1117/1.JBO.19.7.077005 25036216

[B27] Kanoh S.-I. Murayama Y.-M. Miyamoto K.-I. Yoshinobu T. Kawashima R. (2009). “A NIRS-based brain-computer interface system during motor imagery: system development and online feedback training,” in *31st Annual International Conference of the IEEE Engineering in Medicine and Biology Society (EMBC)*, (Minneapolis), 594–597. 10.1109/IEMBS.2009.5333710 19964231

[B28] Kim J. M. Choi J. K. Choi M. Ji M. Hwang G. Ko S. B. (2018). Assessment of cerebral autoregulation using continuous-wave near-infrared spectroscopy during squat-stand maneuvers in subjects with symptoms of orthostatic intolerance. *Sci. Rep.* 8:13257. 10.1038/s41598-018-31685-y 30185974 PMC6125591

[B29] Kwon H. Kim K. Jo Y. Park M. Ko S.-B. Kim T. (2018). Early detection of cerebral infarction with middle cerebral artery occlusion with functional near-infrared spectroscopy: a pilot study. *Front. Neurol.* 9:898. 10.3389/fneur.2018.00898 30467489 PMC6236112

[B30] Lancia S. Choi J. Baek J. Mammarella S. Bianco D. Quaresima V. (2018). “Trail making test induces prefrontal cortex activation as revealed by a cw wearable-wireless fNIRS/DOT imager,” in *Oxygen Transport to Tissue XL*, (Berlin: Springer), 139–144. 10.1007/978-3-319-91287-5_22 30178336

[B31] Lawhern V. J. Solon A. J. Waytowich N. R. Gordon S. M. Hung C. P. Lance B. J. (2018). EEGNet: a compact convolutional neural network for EEG-based brain-computer interfaces. *J. Neural Eng.* 15:056013. 10.1088/1741-2552/aace8c 29932424

[B32] Lebedev M. A. Nicolelis M. A. L. (2006). Brain–machine interfaces: past, present and future. *Trends Neurosci.* 29 536–546. 16859758 10.1016/j.tins.2006.07.004

[B33] Ledoit O. Wolf M. (2004). A well-conditioned estimator for large-dimensional covariance matrices. *J. Multivar. Anal.* 88 365–411.

[B34] Lemm S. Blankertz B. Dickhaus T. Müller K.-R. (2011). Introduction to machine learning for brain imaging. *Neuroimage* 56 387–399. 10.1016/j.neuroimage.2010.11.004 21172442

[B35] Matthews F. Pearlmutter B. A. Ward T. E. Soraghan C. Markham C. (2008). Hemodynamics for brain-computer interfaces. *IEEE Signal Process. Mag.* 25 87–94.

[B36] Mayr A. Binder H. Gefeller O. Schmid M. (2014). The Evolution of Boosting Algorithms From Machine Learning to Statistical Modelling. *Methods Inf. Med.* 53 419–427. 10.3414/ME13-01-0122 25112367

[B37] Mockus J. (2012). *Bayesian Approach to Global Optimization: Theory and Applications.* Berlin: Springer Science & Business Media.

[B38] Nagel S. Spüler M. (2019). World’s fastest brain-computer interface: combining EEG2Code with deep learning. *PLoS One* 14:e0221909. 10.1371/journal.pone.0221909 31490999 PMC6730910

[B39] Naseer N. Hong K.-S. (2015). fNIRS-based brain-computer interfaces: a review. *Front. Hum. Neurosci.* 9:00003. 10.3389/fnhum.2015.00003 PMC4309034 25674060

[B40] Naseer N. Noori F. M. Qureshi N. K. Hong K.-S. (2016). Determining optimal feature-combination for LDA classification of functional near-infrared spectroscopy signals in brain-computer interface application. *Front. Hum. Neurosci.* 10:237. 10.3389/fnhum.2016.00237 27252637 PMC4879140

[B41] Nicholas W. Vernon J. L. Javier O. G. Jennifer C. Josef F. Paul S. (2018). Compact convolutional neural networks for classification of asynchronous steady-state visual evoked potentials. *J. Neural Eng.* 15:066031. 10.1088/1741-2552/aae5d8 30279309

[B42] Nicolas-Alonso L. Gomez-Gil J. (2012). Brain computer interfaces, a review. *Sensors* 12 1211–1279. 10.3390/s120201211 22438708 PMC3304110

[B44] Power S. D. Kushki A. Chau T. (2011). Towards a system-paced near-infrared spectroscopy brain–computer interface: differentiating prefrontal activity due to mental arithmetic and mental singing from the no-control state. *J. Neural Eng.* 8:066004. 10.1088/1741-2560/8/6/066004 21975364

[B43] Power S. D. Kushki A. Chau T. (2012a). Automatic single-trial discrimination of mental arithmetic, mental singing and the no-control state from prefrontal activity: toward a three-state NIRS-BCI. *BMC Res. Notes* 5:141. 10.1186/1756-0500-5-141 22414111 PMC3359174

[B45] Power S. D. Kushki A. Chau T. (2012b). Intersession consistency of single-trial classification of the prefrontal response to mental arithmetic and the no-control state by NIRS. *PLoS One* 7:0037791. 10.1371/journal.pone.0037791 22844390 PMC3402505

[B46] Rakotomarnonjy A. Guigue V. (2008). BCI competition III: dataset II - ensemble of SVMs for BCI P300 speller. *IEEE Trans. Biomed. Eng.* 55 1147–1154. 10.1109/TBME.2008.915728 18334407

[B47] Sakhavi S. Guan C. T. Yan S. C. (2018). Learning temporal information for brain-computer interface using convolutional neural networks. *IEEE Trans. Neural Netw. Learn. Syst.* 29 5619–5629. 10.1109/TNNLS.2018.2789927 29994075

[B48] Schäfer J. Strimmer K. (2005). A Shrinkage approach to large-scale covariance matrix estimation and implications for functional genomics. *Stat. Appl. Genet. Mol. Biol.* 4 1175–1189. 16646851 10.2202/1544-6115.1175

[B49] Schalk G. Mcfarland D. J. Hinterberger T. Birbaumer N. Wolpaw J. R. (2004). BCI2000: A general-purpose, brain-computer interface (BCI) system. *IEEE Trans. Biomed. Eng.* 51 1034–1043. 15188875 10.1109/TBME.2004.827072

[B50] Schudlo L. C. Chau T. (2014). Dynamic topographical pattern classification of multichannel prefrontal NIRS signals: II. Online differentiation of mental arithmetic and rest. *J. Neural. Eng.* 11:016003. 10.1088/1741-2560/11/1/016003 24311057

[B51] Schudlo L. C. Chau T. (2015). Towards a ternary NIRS-BCI: single-trial classification of verbal fluency task. Stroop task and unconstrained rest. *J. Neural Eng.* 12:066008. 10.1088/1741-2560/12/6/066008 26447770

[B52] Sereshkeh A. R. Yousefi R. Wong A. T. Chau T. (2019). Online classification of imagined speech using functional near-infrared spectroscopy signals. *J. Neural Eng.* 16:016005. 10.1088/1741-2552/aae4b9 30260320

[B53] Shin J. Kwon J. Choi J. Im C.-H. (2017a). Performance enhancement of a brain-computer interface using high-density multi-distance NIRS. *Sci. Rep.* 7:16545. 10.1038/s41598-017-16639-0 29185494 PMC5707382

[B54] Shin J. Kwon J. Choi J. Im C. H. (2018a). Ternary near-infrared spectroscopy brain-computer interface with increased information transfer rate using prefrontal hemodynamic changes during mental arithmetic, breath-Holding, and idle State. *IEEE Access* 6 19491–19498.

[B55] Shin J. Kwon J. Im C.-H. (2018b). A ternary hybrid EEG-NIRS brain-computer interface for the classification of brain activation patterns during mental arithmetic, motor imagery, and idle state. *Front. Neuroinform.* 12:5. 10.3389/fninf.2018.00005 29527160 PMC5829061

[B58] Shin J. Müller K.-R. Hwang H.-J. (2016). Near-infrared spectroscopy (NIRS) based eyes-closed brain-computer interface (BCI) using prefrontal cortex activation due to mental arithmetic. *Sci. Rep.* 6:36203. 10.1038/srep36203 27824089 PMC5099935

[B56] Shin J. Von Lühmann A. Blankertz B. Kim D.-W. Jeong J. Hwang H.-J. (2017b). Open access dataset for EEG+NIRS single-trial classification. *IEEE Trans. Neural Syst. Rehabil. Eng.* 25 1735–1745. 10.1109/TNSRE.2016.2628057 27849545

[B57] Shin J. Von Lühmann A. Kim D.-W. Mehnert J. Hwang H.-J. Müller K.-R. (2018c). Simultaneous acquisition of EEG and NIRS during cognitive tasks for an open access dataset. *Sci. Data* 5:180003. 10.1038/sdata.2018.3 29437166 PMC5810421

[B59] Sun S. L. Zhang C. S. Zhang D. (2007). An experimental evaluation of ensemble methods for EEG signal classification. *Pattern Recognit. Lett.* 28 2157–2163. 10.1109/TNSRE.2015.2496334 26552089

[B60] Trakoolwilaiwan T. Behboodi B. Lee J. Kim K. Choi J. W. (2018). Convolutional neural network for high-accuracy functional near-infrared spectroscopy in a brain-computer interface: three-class classification of rest, right-, and left-hand motor execution. *Neurophotonics* 5:011008. 10.1117/1.NPh.5.1.011008 28924568 PMC5599227

[B61] Vidaurre C. Blankertz B. (2010). Towards a cure for BCI illiteracy. *Brain Topogr.* 23 194–198. 10.1007/s10548-009-0121-6 19946737 PMC2874052

[B62] Wolpaw J. R. Birbaumer N. Mcfarland D. J. Pfurtscheller G. Vaughan T. M. (2002). Brain-computer interfaces for communication and control. *Clin. Neurophysiol.* 113 767–791. 12048038 10.1016/s1388-2457(02)00057-3

[B63] Xiao Y. W. Wu J. Lin Z. L. Zhao X. D. (2018). A deep learning-based multi-model ensemble method for cancer prediction. *Comput. Methods Programs Biomed.* 153 1–9. 10.1016/j.cmpb.2017.09.005 29157442

[B64] Zafar A. Hong K.-S. (2017). Detection and classification of three-class initial dips from prefrontal cortex. *Biomed. Opt. Express* 8 367–383. 10.1364/BOE.8.000367 28101424 PMC5231305

