# Supplementary material for: Performance Improvement of Near-Infrared Spectroscopy-Based Brain-Computer Interface Using Regularized Linear Discriminant Analysis Ensemble Classifier Based on Bootstrap Aggregating
Source: Front Neurosci. 2020 Mar 4;14:168. doi: 10.3389/fnins.2020.00168 (PMC7064639; doi:10.3389/fnins.2020.00168)
Supplement: Supplementary file 1 [file Data_Sheet_1.PDF]

Supplementary Information File

Performance improvement of near-infrared spectroscopy-based brain-computer interface using regularized linear discriminant analysis ensemble classifier based on bootstrap aggregating

Jaeyoung Shin[1] and Chang-Hwan Im[2*]

[1]Department of electronic engineering, Wonkwang University, Iksan, Korea
[2]Department of biomedical engineering, Hanyang University, Seoul, Korea

In this supplementary information file, we provide individual classification accuracies computed by three types of strong learners (linear support vector machine (SVM), linear discriminant analysis (LDA), and regularized linear discriminant analysis (RLDA)) and the bootstrap aggregating ensemble learning (bagging). Tables SI-SIV show the individual classification accuracies for datasets I-IV. Bitrates based on individual classification accuracies are shown in Tables SV-SVIII.

Table SI. Individual classification accuracies estimated by three types of strong learners and the bagging ensemble learning: dataset I.

Participant	SVM	LDA	RLDA	Bagging
1	37.0	41.7	30.0	38.7
2	60.7	61.5	59.0	65.5
3	55.5	57.0	54.8	60.5
4	63.2	55.2	64.8	65.7
5	62.7	67.3	60.0	65.8
6	52.3	56.0	55.2	66.0
7	45.7	48.8	45.3	50.7
8	52.5	47.3	54.0	51.7
9	73.5	58.0	71.3	72.7
10	50.7	51.5	45.5	52.2
11	60.5	53.0	49.2	62.7
12	47.7	49.5	60.2	55.7
13	74.7	77.0	72.7	78.0
14	64.7	57.8	62.0	68.7
15	59.7	54.7	68.7	66.7
16	64.8	73.8	68.0	69.2
17	55.7	63.7	54.0	56.8
18	61.7	56.0	61.8	57.2
19	65.5	63.8	57.8	70.2
20	67.2	70.8	78.3	73.8
21	75.5	73.0	63.2	76.8
22	50.2	51.2	50.2	57.2
23	68.7	60.5	71.5	68.0
24	45.5	35.0	28.5	41.5
25	67.2	60.3	62.3	67.7
26	68.0	64.0	65.5	67.2
27	68.5	63.5	69.5	70.3
28	56.2	57.8	64.5	62.5
29	53.3	49.3	65.5	56.3
**mean**	**59.6**	**57.9**	**59.1**	**62.6**
**std**	**9.5**	**9.5**	**11.6**	**9.6**

Table SII. Individual classification accuracies estimated by three types of strong learners and the bagging ensemble learning: dataset II.

Participant	SVM	LDA	RLDA	Bagging
1	87.5	83.3	90.0	89.2
2	88.5	91.8	94.7	92.3
3	83.0	77.3	92.5	83.0
4	79.7	75.8	78.7	89.2
5	81.8	74.7	76.7	83.5
6	94.0	91.3	90.3	94.7
7	85.8	76.5	88.7	89.3
8	93.2	93.7	95.0	95.5
9	83.7	83.3	87.7	90.2
10	97.8	97.3	99.2	98.3
11	87.2	80.0	87.2	90.0
12	83.0	84.8	81.3	89.5
13	85.5	85.5	78.8	89.2
14	86.5	83.5	84.2	88.8
15	73.0	71.2	74.0	80.7
16	82.3	76.3	85.3	81.7
17	91.7	88.2	94.8	92.5
18	98.3	99.0	99.3	99.0
19	86.2	75.0	70.8	81.5
20	67.2	65.5	68.3	66.2
21	91.3	90.7	94.0	93.3
22	80.7	92.5	90.8	91.7
23	65.8	68.8	72.8	70.0
24	78.7	81.2	81.2	82.2
25	86.2	83.8	92.7	91.7
26	85.7	77.5	90.7	86.5
27	99.2	83.5	90.3	98.0
28	93.3	94.7	87.5	95.8
29	89.7	88.3	96.8	94.3
**mean**	**85.7**	**83.3**	**86.7**	**88.5**
**std**	**8.1**	**8.6**	**8.6**	**7.7**

Table SIII. Individual classification accuracies estimated by three types of strong learners and the bagging ensemble learning: dataset III.

Participant	SVM	LDA	RLDA	Bagging
1	77.2	79.3	67.3	73.7
2	75.2	67.7	82.8	88.7
3	87.7	86.2	84.5	85.2
4	65.0	64.0	66.7	68.5
5	84.2	83.5	84.7	85.8
6	70.8	75.5	61.3	63.0
7	83.2	87.0	78.0	78.8
8	58.5	60.3	44.8	45.7
9	72.0	68.7	76.7	74.5
10	76.8	70.3	69.0	72.8
11	59.2	68.3	58.2	61.0
12	76.2	73.8	48.3	51.8
13	66.8	67.8	68.7	70.0
14	75.8	79.7	83.7	85.0
15	57.8	62.8	72.8	74.2
16	93.2	91.8	94.5	94.8
17	75.0	76.0	70.0	74.5
18	75.0	71.8	57.0	62.3
19	71.0	67.5	79.3	82.8
20	72.3	55.8	60.5	68.7
21	55.3	55.5	57.3	60.3
22	76.8	74.3	64.0	75.2
23	67.0	66.2	72.2	73.5
24	79.7	76.0	82.0	80.0
25	87.2	81.3	84.0	88.3
26	80.0	72.3	82.3	84.0
**mean**	**73.8**	**72.4**	**71.2**	**74.0**
**std**	**9.6**	**9.2**	**12.4**	**11.8**

Table SIV. Individual classification accuracies estimated by three types of strong learners and the bagging ensemble learning: dataset IV.

Participant	SVM	LDA	RLDA	Bagging
1	68.1	69.2	72.6	74.2
2	68.8	69.4	69.1	71.1
3	49.8	46.9	54.2	56.7
4	78.8	79.9	79.2	82.1
5	54.7	55.7	49.4	58.6
6	64.8	64.9	76.4	77.7
7	44.0	43.1	48.3	46.3
8	59.7	59.2	58.4	59.4
9	58.0	55.1	64.7	65.2
10	60.2	61.0	68.0	70.0
11	64.0	66.2	68.1	66.7
12	65.8	64.1	66.0	69.1
13	74.4	71.4	78.9	77.6
14	68.6	67.7	69.1	75.1
15	84.8	85.4	89.0	89.1
16	58.8	58.7	59.0	60.7
17	64.9	64.7	67.4	68.0
**mean**	**64.0**	**63.7**	**66.9**	**68.7**
**std**	**10.0**	**10.5**	**10.8**	**10.5**

Table SV. Individual bitrates based on the classification accuracies estimated by three types of strong learners and the bagging ensemble learning: dataset I.

Participant	SVM	LDA	RLDA	Bagging
1	0.00	0.00	0.00	0.00
2	0.20	0.23	0.14	0.42
3	0.05	0.09	0.04	0.19
4	0.30	0.05	0.39	0.43
5	0.28	0.53	0.17	0.44
6	0.01	0.06	0.05	0.45
7	0.00	0.00	0.00	0.00
8	0.01	0.00	0.03	0.00
9	0.99	0.11	0.81	0.92
10	0.00	0.00	0.00	0.01
11	0.19	0.02	0.00	0.28
12	0.00	0.00	0.18	0.06
13	1.10	1.33	0.92	1.44
14	0.38	0.11	0.25	0.62
15	0.16	0.04	0.62	0.49
16	0.39	1.02	0.57	0.65
17	0.06	0.33	0.03	0.08
18	0.24	0.06	0.24	0.09
19	0.42	0.34	0.11	0.72
20	0.52	0.77	1.48	1.02
21	1.18	0.95	0.30	1.31
22	0.00	0.00	0.00	0.09
23	0.62	0.19	0.83	0.57
24	0.00	0.00	0.00	0.00
25	0.52	0.19	0.27	0.55
26	0.57	0.34	0.42	0.52
27	0.61	0.32	0.68	0.74
28	0.07	0.11	0.37	0.27
29	0.02	0.00	0.42	0.07
**mean**	**0.31**	**0.25**	**0.32**	**0.43**
**std**	**0.34**	**0.35**	**0.36**	**0.39**

Table SVI. Individual bitrates based on the classification accuracies estimated by three types of strong learners and the bagging ensemble learning: dataset II.

Participant	SVM	LDA	RLDA	Bagging
1	2.74	2.10	3.19	3.03
2	2.91	3.55	4.20	3.66
3	2.05	1.37	3.69	2.05
4	1.63	1.21	1.51	3.03
5	1.90	1.10	1.30	2.12
6	4.04	3.45	3.25	4.20
7	2.47	1.28	2.94	3.06
8	3.84	3.96	4.28	4.41
9	2.15	2.10	2.77	3.22
10	5.10	4.94	5.58	5.27
11	2.68	1.67	2.68	3.19
12	2.05	2.32	1.83	3.09
13	2.42	2.42	1.53	3.03
14	2.57	2.12	2.22	2.97
15	0.95	0.80	1.04	1.75
16	1.96	1.26	2.39	1.88
17	3.52	2.85	4.24	3.69
18	5.27	5.52	5.65	5.52
19	2.52	1.13	0.77	1.85
20	0.52	0.42	0.60	0.46
21	3.45	3.32	4.04	3.88
22	1.75	3.69	3.35	3.52
23	0.44	0.63	0.94	0.71
24	1.51	1.81	1.81	1.94
25	2.52	2.17	3.73	3.52
26	2.44	1.38	3.32	2.57
27	5.58	2.12	3.25	5.15
28	3.88	4.20	2.74	4.50
29	3.12	2.88	4.78	4.12
**mean**	**2.69**	**2.34**	**2.88**	**3.15**
**std**	**1.28**	**1.30**	**1.38**	**1.25**

Table SVII. Individual bitrates based on the classification accuracies estimated by three types of strong learners and the bagging ensemble learning: dataset III.

Participant	SVM	LDA	RLDA	Bagging
1	1.35	1.59	0.53	1.01
2	1.15	0.55	2.03	2.94
3	2.77	2.52	2.27	2.37
4	0.40	0.34	0.49	0.61
5	2.22	2.12	2.29	2.47
6	0.77	1.18	0.22	0.30
7	2.08	2.66	1.44	1.53
8	0.13	0.19	0.00	0.00
9	0.87	0.62	1.30	1.09
10	1.31	0.74	0.64	0.94
11	0.15	0.60	0.12	0.21
12	1.25	1.02	0.00	0.01
13	0.50	0.56	0.62	0.71
14	1.21	1.63	2.15	2.34
15	0.11	0.29	0.94	1.05
16	3.84	3.55	4.16	4.24
17	1.13	1.23	0.71	1.09
18	1.13	0.85	0.09	0.27
19	0.79	0.54	1.59	2.03
20	0.89	0.06	0.19	0.62
21	0.05	0.05	0.09	0.19
22	1.31	1.07	0.34	1.15
23	0.51	0.46	0.88	0.99
24	1.63	1.23	1.92	1.67
25	2.68	1.83	2.19	2.88
26	1.67	0.89	1.96	2.19
**mean**	**1.23**	**1.09**	**1.12**	**1.34**
**std**	**0.91**	**0.87**	**1.02**	**1.07**

Table SVIII. Individual bitrates based on the classification accuracies estimated by three types of strong learners and the bagging ensemble learning: dataset IV.

Participant	SVM	LDA	RLDA	Bagging
1	2.18	2.32	2.78	3.02
2	2.26	2.35	2.31	2.57
3	0.50	0.34	0.79	0.99
4	3.76	3.96	3.84	4.37
5	0.83	0.91	0.48	1.15
6	1.78	1.79	3.37	3.57
7	0.21	0.18	0.41	0.31
8	1.25	1.21	1.14	1.23
9	1.10	0.86	1.77	1.83
10	1.31	1.38	2.16	2.42
11	1.69	1.95	2.18	2.00
12	1.89	1.71	1.92	2.31
13	3.06	2.62	3.78	3.55
14	2.23	2.12	2.31	3.16
15	4.90	5.04	5.85	5.88
16	1.17	1.16	1.19	1.35
17	1.79	1.77	2.09	2.16
**mean**	**1.88**	**1.86**	**2.26**	**2.46**
**std**	**1.18**	**1.22**	**1.39**	**1.38**
